# Multimodal cross-attention network for overgrowth detection in strawberry seedlings

**DOI:** 10.3389/fpls.2025.1706694

**Published:** 2026-01-02

**Authors:** Zhenzhen Cheng, Yifan Cheng, Tingting Fang, Man Zhu, Jing Liu, Peng Qi, Qiaoyu Zhang

**Affiliations:** 1 Department of Horticulture, Xinyang Agriculture and Forestry University, Xinyang, China; 2 Department of Optical and Electronic Information, Huazhong University of Science and Technology, Wuhan, China; 3 Shandong Academy of Agricultural Machinery Sciences (SAAMS), Jinan, China; 4 Centre for Agricultural Robotics Innovation (CARI), Jinan, China

**Keywords:** strawberry, overgrowth, multimodal fusion, cross-attention, early warning

## Abstract

Early warning of overgrowth in strawberry seedlings is essential to balance vegetative and reproductive growth. However, existing monitoring methods face major challenges, including subtle visual symptoms and limited abnormal samples. To address this, we propose MM-CAPNet, a multimodal fusion framework for early detection of seedling overgrowth. We first developed a representative sample collection of strawberry seedlings through a systematic induction experiment, integrating historical environmental time-series data with contemporaneous plant images. The MM-CAPNet architecture uses a dual-stream design to process these inputs, with a Transformer encoder for environmental sequences and a MobileNetV2 encoder for images. A critical component of the proposed framework lies in the image-guided Cross-Attention mechanism, which uniquely treats the current phenotype as an active query to adaptively retrieve and aggregate the most diagnostically relevant segments of past environmental data. Experiments show MM-CAPNet outperforms baselines, reaching 87.6% accuracy and 0.901 AUC, with strong discriminative ability for early overgrowth categories. Ablation studies confirm its interpretability by linking visual phenotypes to key environmental drivers. This work provides growers with a proof-of-concept framework to regulate fertilization, irrigation, and light management during the nursery stage, thereby reducing the risk of excessive vegetative growth. The proposed framework supports precision cultivation strategies that enhance resource efficiency and crop resilience.

## 1 Introduction

Strawberry, a high-value horticultural crop, is highly sensitive to growth conditions during the seedling stage, which strongly determines subsequent yield and fruit quality (Cockerton et al., 2021; Fagherazzi et al., 2021; Lekavičienė et al., 2025). A common challenge in nurseries is excessive vegetative growth, a condition referred to as overgrowth, which is manifested as enlarged leaves and elongated stems (Katel et al., 2023). This impedes flower bud initiation, reduces fruit set, and lowers fruit quality (Hwang et al., 2020; Ndikumana et al., 2025). Therefore, the ability to accurately identify and provide early warning of overgrowth at the seedling stage is pivotal for precision cultivation and intelligent management.

Traditional approaches to crop vigor assessment include manual observation, empirical growth modeling, and remote sensing (Wang et al., 2019). Manual observation depends on growers’ subjective judgment and is poorly reproducible. Even under a unified protocol, vigor scores varied widely among observers (Smith et al., 2023). Physiological models can predict biomass in crops such as rice, maize, and wheat, but their reliance on empirical assumptions limits real-time monitoring of subtle growth shifts in strawberry seedlings (Palosuo et al., 2011; Shimono et al., 2023; Winn et al., 2023). While remote sensing techniques (e.g., NDVI and related vegetation indices) offer non-invasive monitoring, in nursery settings they suffer from insufficient resolution, inability to capture subtle morphological cues (e.g., leaf curling, angle variation), and poor adaptability to greenhouse conditions (Huete et al., 2002; Jiménez-Lao et al., 2020; Wu et al., 2023). Overall, current methods lack objectivity, timeliness, and practicality in nursery settings.

Modern sensing technologies provide the means to capture rich visual and environmental data, but extracting predictive patterns requires advanced analytical tools. Artificial intelligence (AI), has therefore become central to crop phenotyping owing to its strong feature representation capacity (Pound et al., 2017; Singh et al., 2018; Gill et al., 2022). Convolutional neural networks (CNNs) have been extensively applied in crop imaging tasks, effectively extracting morphological and color features for early stress detection in tomato, cabbage, and other crops (Yang and Xu, 2021; Zhu et al., 2022; Lin et al., 2024). With growing recognition of crop growth as a dynamic process, time-series models such as long short-term memory (LSTM) networks and Transformers have been adopted to capture the temporal evolution of environmental factors (Jiang et al., 2018; Ibañez and Monterola, 2023). Despite their effectiveness, the limitations of unimodal inputs are increasingly evident, as crop growth is not solely determined by current visual phenotypes but is shaped by the cumulative effects of past environmental conditions and future climatic fluctuations (Li et al., 2024; Shamsuddin et al., 2024). This understanding has driven research toward multimodal data integration, particularly combining imagery with environmental sensor data, to achieve synergistic perception. Studies have already explored combining CNNs with LSTM or Transformer models for yield prediction and stress monitoring in maize, wheat, and soybean, with promising results. For example, a CNN–RNN framework that learns spatiotemporal features from environmental and management factors achieved substantially better performance than traditional models in maize and soybean yield prediction, with prediction errors of only 9% and 8% of the average yield, respectively (Khaki et al., 2019). In addition, a dual-branch CNN–LSTM model integrating remote sensing and meteorological data for winter wheat yield prediction demonstrated significantly higher accuracy compared with methods that rely solely on CNN or LSTM (Zhang et al., 2024).

While these advances highlight the potential of multimodal deep learning in agriculture, most applications remain focused on field crops with relatively uniform distributions. By contrast, strawberry seedlings exhibit compact, subtle, and rapidly changing phenotypes, requiring higher precision and timeliness in monitoring (Ndikumana et al., 2024). Several representative studies have reported how specific environmental factors regulate the delicate balance between vegetative and reproductive growth in strawberry seedlings. Light intensity and quality critically influence flower initiation and runner production; reduced light or shading delays floral differentiation and promotes excessive vegetative growth (Wang et al., 2020). Night temperature and photoperiod exert strong control over flowering responses across cultivars (Sønsteby and Heide, 2008). Irrigation and soil moisture levels significantly affect canopy expansion and root development, thereby modulating seedling vigor under protected cultivation (Pires et al., 2006). Moreover, developmental stage and chilling sensitivity determine narrow temporal windows during which floral buds form and are particularly vulnerable to environmental perturbations (Ariza et al., 2015). These representative cases underline the narrow time scales and subtle phenotypic signals characteristic of strawberry seedlings. Consequently, applying existing techniques to the early warning of strawberry overgrowth faces three major challenges. First is the challenge of timeliness in recognition. Most existing studies have focused on detecting abnormal states once visual symptoms have become apparent, such as lesion identification or fruit counting (Mahlein, 2016; Vasconez et al., 2020). However, for facility-grown crops such as strawberries, the most valuable intervention often lies in the incipient or presymptomatic stage, when visual cues are still negligible. The real challenge, therefore, is to overcome the limitations of relying solely on visible light imaging and to shift from *post hoc* diagnosis to early warning. This requires leveraging high-dimensional data to capture subtle physiological changes in crops, thereby enabling highly sensitive pre-diagnosis of overgrowth (Rumpf et al., 2010; Li et al., 2022). Second is the challenge of multimodal fusion and cross-source complementarity. Integrating image-based phenotypic data with environmental time-series data to build more robust predictive models has become a widely shared consensus in the field (Liao et al., 2024; Yang et al., 2024; Liu et al., 2025). Yet, this consensus conceals a substantial technological gap. Images and temporal data are inherently heterogeneous in terms of data structure, information density, and feature dimensionality. Designing advanced fusion mechanisms to address this heterogeneity remains an open problem (Meng et al., 2020; Maillet et al., 2023). An ideal fusion model should not only accommodate these structural differences but also capture the early and subtle nonlinear correlations across modalities that signal the onset of overgrowth. Finally is the challenge of data scarcity and model training. In controlled facility environments, abnormal or transitional phases such as seedling overgrowth occur as low-frequency events. This scarcity, together with the resulting class imbalance, hampers both the training and generalization ability of deep learning models (Krawczyk, 2016; Kamilaris and Prenafeta-Boldú, 2018). Systematic solutions to the pervasive issue of sparse samples in real-world agricultural scenarios, particularly in strawberry growth prediction, have not yet received sufficient attention or resolution (Lee et al., 2022; Aghamohammadesmaeilketabforoosh et al., 2024).

To overcome the limitations of existing approaches, this study makes the following contributions: (1) A cross-temporal heterogeneous dataset was constructed by integrating historical environmental sequences with contemporaneous seedling images, enabling a transition from mere recognition to early warning of strawberry overgrowth. (2) MM-CAPNet, a dual-stream multimodal fusion model equipped with an image-guided Cross-Attention mechanism, was designed to adaptively align and amplify critical information across modalities, capturing subtle, early-stage overgrowth patterns. (3) A systematic overgrowth induction protocol was developed to generate abundant, balanced, and diverse samples under controlled conditions, providing robust support for model training and a transferable methodological framework for phenotyping studies. These innovations establish a robust methodological advance toward precision seedling vigor monitoring and sustainable strawberry production.

## 2 Materials and methods

### 2.1 Dataset and preprocessing

**Figure 1:**
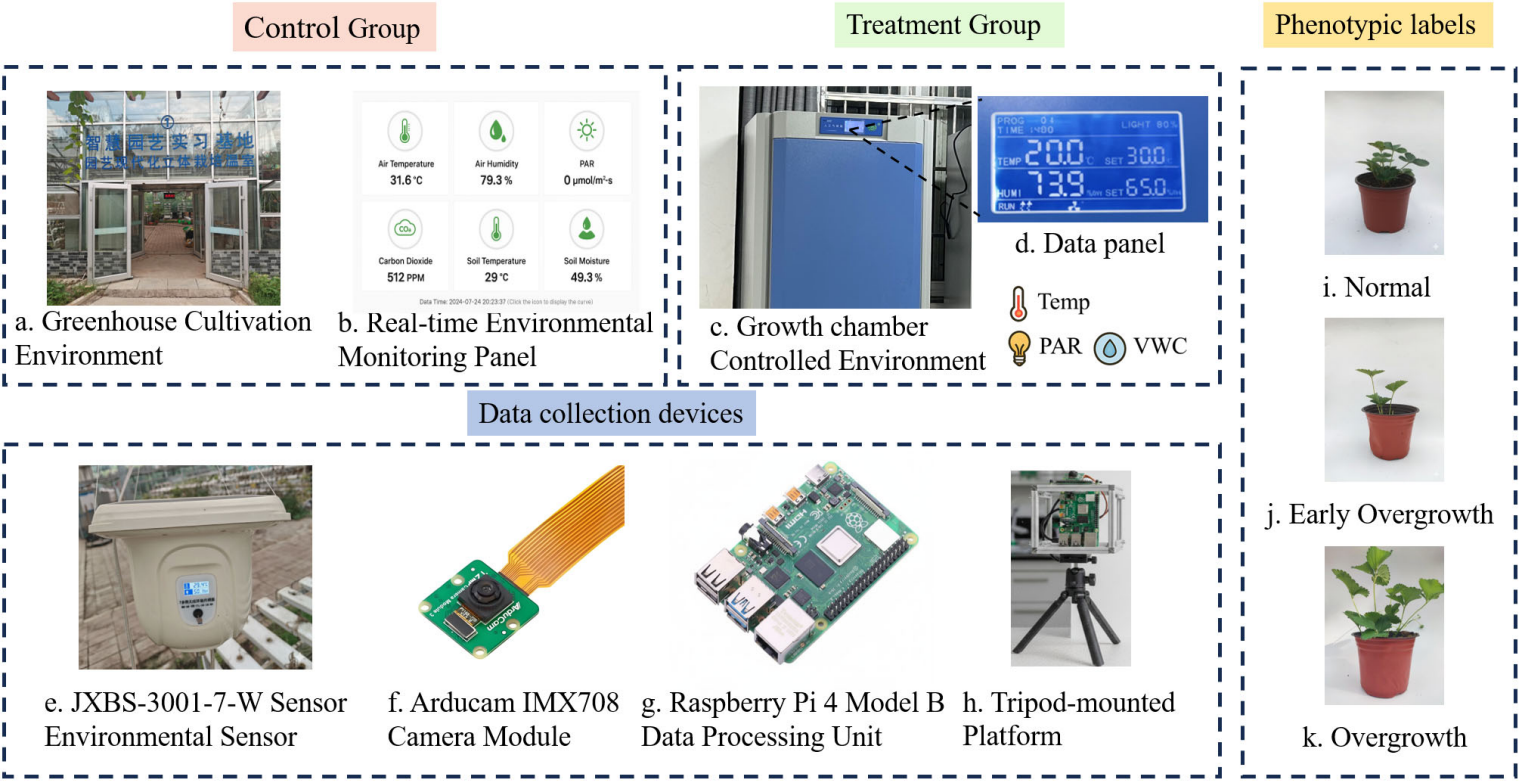
Experimental environment, data collection, and phenotypic labels. **(a–d)** Experimental environment; **(e–h)** Collection equipment; **(i–k)** Phenotypic labeling.

The experiment was conducted at the College of Horticulture, Xinyang Agriculture and Forestry University, Henan Province, China, using the strawberry cultivar ‘Benihoppe’. All seedlings were cultivated in pots of uniform specifications. To construct a multimodal dataset covering the full gradient from normal growth to varying degrees of overgrowth, we designed a dual-group experimental system consisting of a control group and a treatment group (**Figures 1A–D**).

The Control Group was grown under standard cultivation conditions in an intelligent greenhouse, where plants were primarily influenced by natural weather fluctuations. This group was intended to record the baseline growth process of strawberry seedlings under conventional management and to provide samples representing the normal state for the model. In contrast, the Treatment Group was designed to overcome the inherent scarcity of overgrowth samples in natural environments by inducing excessive growth through controlled interventions. At designated developmental stages, seedlings were transferred into a growth chamber (Yiheng, Shanghai, China) equipped with precise, independent control of temperature, Photosynthetically Active Radiation (PAR), and photoperiod.

Three induction modes were applied: temperature, PAR, and Soil volumetric water content (VWC), each at two intensity levels (Low/High). Prior to treatment, all seedlings underwent a 7-day preadaptation period under a standardized baseline environment, calibrated in preliminary trials: night temperature (Temp_n_) was set to 10 °C; baseline PAR (PAR_0_) was maintained at 400 μmol·m^-^²·s^-^¹; photoperiod was fixed at 12 h light/12 h dark; and baseline VWC (VWC_0_) was maintained at 75% of field capacity. Each treatment was then applied for 10 consecutive days, after which plants were returned to conventional management. This 10-day duration was chosen based on preliminary trials, where strawberry seedlings showed measurable morphological responses (e.g., leaf expansion, petiole elongation) within 7–12 days under stress conditions. This duration balances inducing visible physiological changes with avoiding severe or irreversible stress, consistent with prior studies indicating that 7–14 days of environmental fluctuations during the seedling stage suffice to reveal significant morphological changes characteristic of overgrowth (Grant et al., 2010; Bo et al., 2024; Chu et al., 2024). Treatment intensities relative to baseline conditions were defined as follows: (1) Temperature: Low treatment corresponded to an increase of 2°C above Temp_n_, resulting in a night temperature of 12°C; High treatment corresponded to an increase of 5°C above Temp_n_, resulting in a night temperature of 15°C. Daytime temperature was maintained constant in both cases. (2) PAR: Low treatment corresponded to 65% of PAR_0_ (260 μmol·m^-^²·s^-^¹), and High treatment corresponded to 35% of PAR_0_ (140 μmol·m^-^²·s^-^¹), with the photoperiod maintained but photosynthetic photon flux density adjusted accordingly. (3) VWC: Low treatment corresponded to an increase of 7% relative to VWC_0_, resulting in a VWC of approximately 82% of field capacity; High treatment corresponded to an increase of 12% relative to VWC_0_, resulting in approximately 87% of field capacity. These adjustments were achieved by modifying irrigation frequency and volume while avoiding waterlogging.

Plants exhibiting severe pathological symptoms (e.g., extensive chlorosis, necrosis, or wilting) during the treatment period were terminated early according to a predefined stopping rule, and the cause was recorded. To ensure statistical power and class representativeness, each treatment combination (three induction modes × two intensity levels, resulting in six combinations) included 48 biological replicates (individual potted plants), totaling 288 plants in the treatment group. The control group comprised 96 plants, yielding approximately 384 plants in total (with a contingency plan of 36 replicates per combination if limited by facility capacity). This scale ensured sufficient sample diversity for subsequent partitioning into training, validation, and test sets while minimizing variability among individual plants.

Environmental data (temperature, PAR, VWC) were continuously monitored in both the greenhouse and the growth chamber using a calibrated JXBS-3001-7-W Wireless Seven-Parameter Environmental Sensor (Jingxuntong, Weihai, Shandong, China) (**Figure 1E**). The manufacturer-reported uncertainties are ±0.2 °C for temperature, ± 3 % for PAR, and ±2 % for VWC. Among the seven environmental parameters measured by the JXBS-3001-7-W sensor, three—temperature, photosynthetically active radiation (PAR), and soil volumetric water content (VWC)—were selected as model inputs. These variables were chosen because they represent the dominant, directly controllable environmental drivers that regulate vegetative growth in strawberry seedlings, as supported by prior studies (Wang and Zheng, 2001; Guiamba et al., 2022; Jiang et al., 2022; Petrakis et al., 2024). In contrast, other parameters such as air humidity and CO_2_ concentration exhibited high short-term variability and low correlation with growth morphology (Ahn et al., 2022; Hu et al., 2023). Therefore, these factors were excluded to avoid model overfitting and reduce redundant inputs.

Raw measurements were recorded every 10 minutes and downsampled by averaging within 2-hour windows to reduce high-frequency noise and align with the model’s temporal scale. Each 10-day treatment sequence consisted of 120-time steps, corresponding to 12 samples per day. To capture plant phenotypes, images were collected under controlled conditions: each plant was placed against a fixed white background board, and images were acquired daily at 10:00 a.m. using an integrated device consisting of a Raspberry Pi 4 Model B (Broadcom, San Jose, California, USA) and an Arducam IMX708 camera module (Arducam, Nanjing, Jiangsu Province, China) (**Figures 1F–H**). Raw images (1280 × 720 px) were resized and center-cropped to 224 × 224 px for model input. Phenotypic labels were assigned daily by expert horticulturists into three categories: Normal, Early Overgrowth, and Overgrowth (**Figures 1I–K**). The independent annotators achieved an exact-match inter-annotator agreement of 97% across all labeled samples. Disagreements (3% of cases) were resolved via a consensus discussion among the three experts and a final consensus label was assigned.

To expand the dataset and address class imbalance, a sliding time-window strategy was applied. For each plant, 10-day sequences were generated with a stride of 2 days, producing multiple overlapping samples. The stride length was empirically determined to balance sample volume and redundancy. Specifically, a stride of one day would yield highly correlated samples and impose excessive computational costs, whereas a stride of three days would markedly reduce the number of samples and the diversity of temporal patterns. Accordingly, a stride of two days was adopted as an optimal compromise between dataset expansion and sample independence. Each sample consisted of (1) an environmental time series of 120-time steps with 3 variables each (120 × 3), (2) the RGB image from the last day (224 × 224 px × 3 channels), and (3) the expert-assigned label. This strategy substantially increased sample size, improved temporal diversity, and alleviated the scarcity of early overgrowth cases, thereby enhancing class balance in training.

Data quality control included: interpolating missing sensor values for gaps of up to one time step (2 hours), discarding windows with longer gaps; removing images with severe blurring or occlusion; and excluding early-stopped plants from the final dataset. Repeated use of overlapping windows may introduce statistical dependency among samples; to mitigate this risk, all windows derived from the same plant were strictly assigned to either the training, validation, or test set, thereby preventing information leakage. After cleaning and quality checks, a total of 900 valid samples were obtained, distributed as follows: Normal (302), Early Overgrowth (298), and Overgrowth (300). Stratified sampling was applied to ensure consistent class proportions across subsets, resulting in: Training set (70%): 211 Normal, 209 Early Overgrowth, 210 Overgrowth (total 630 samples); Validation set (15%): 45 Normal, 45 Early Overgrowth, 45 Overgrowth (total 135 samples); Test set (15%): 46 Normal, 44 Early Overgrowth, 45 Overgrowth (total 135 samples).To avoid information leakage arising from overlapping time windows, all samples derived from the same plant were strictly assigned to a single subset (training, validation, or test).

### 2.2 Proposed method

**Figure 2:**
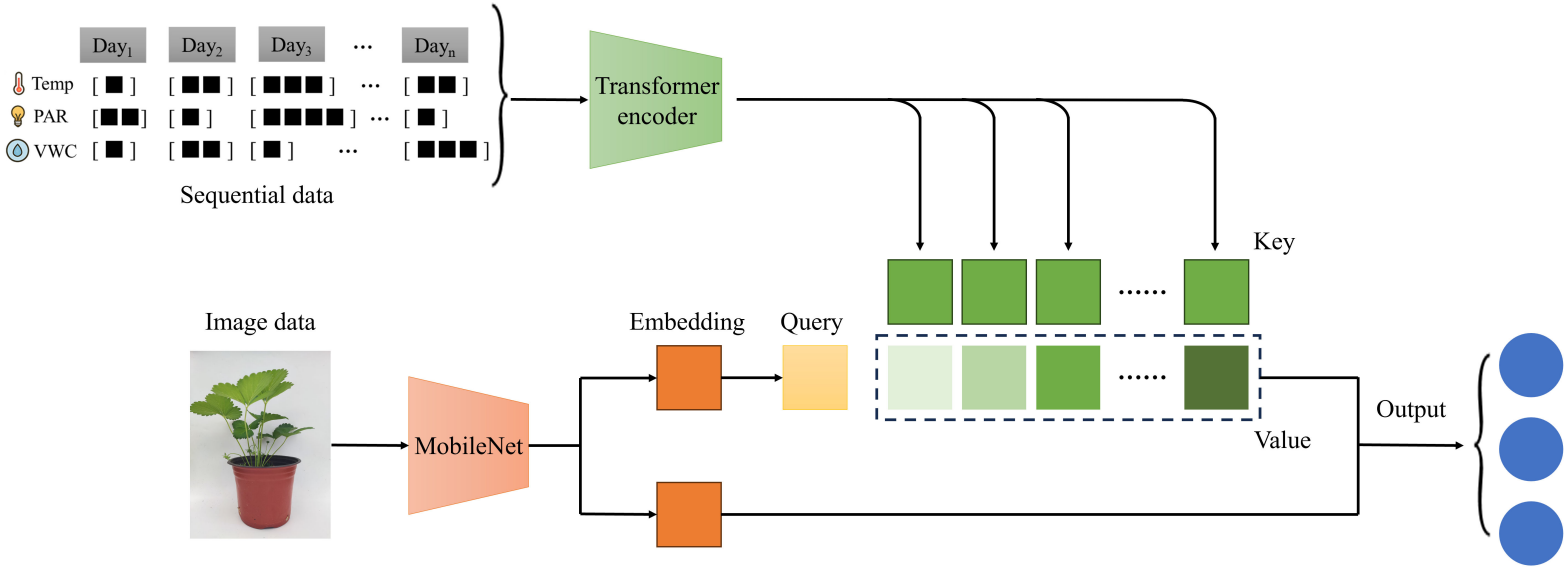
Overall architecture of the proposed MM-CAPNet.

To achieve accurate early identification of overgrowth, we propose MM-CAPNet, a dual-modality fusion framework. The overall architecture, illustrated in **Figure 2**, follows a dual-expert paradigm with an image-guided Cross-Attention fusion mechanism. The network integrates two heterogeneous but complementary data sources: environmental time-series data reflecting historical growth conditions, and single-view RGB images capturing the current phenotype. Specifically, the model comprises two parallel branches. First, the environmental branch processes a sequence of daily data (Temp: Temperature, PAR: Photosynthetically Active Radiation, VWC: Volumetric Water Content) using a Transformer Encoder. This module is designed to capture long-range dependencies within the environmental sequence, encoding the historical context into Key (K) and Value (V) pairs. Second, the vision branch employs a lightweight MobileNetV2 to efficiently extract discriminative plant features from an RGB image. This visual representation is then projected to form a Query (Q) vector. At the fusion stage, we introduce a novel image-guided Cross-Attention mechanism. In this core module, the image-derived Query (Q) selectively attends to the most informative fragments of the historical environmental dynamics, which are represented by the Key-Value (K-V) pairs. This process yields a complementary, semantically enriched representation. Finally, this attention weighted context is concatenated with the original image feature embedding, and the resulting fused vector is fed into a fully connected classifier to predict the plant’s growth state: Normal, Early Overgrowth, or Overgrowth.

#### 2.2.1 Transformer-encoder for environmental sequences

**Figure 3:**
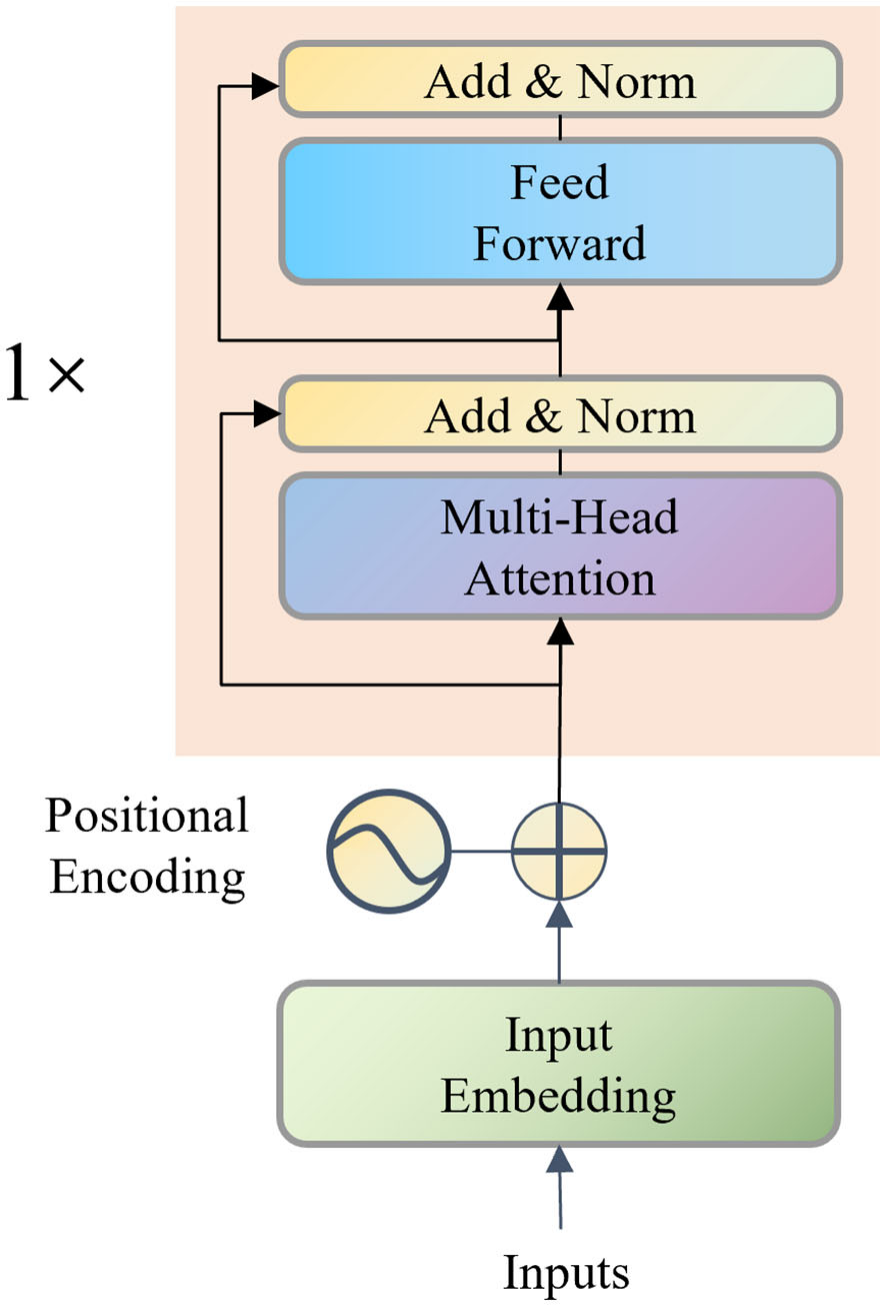
The Transformer-Encoder module.

Accurately characterizing the dynamic evolution of environmental variables is crucial for identifying growth trends driven by cumulative environmental effects. Raw sensor signals are often periodic, delayed, and exhibit long-range dependencies, which recurrent models (e.g., LSTM, GRU) struggle to capture due to gradient vanishing and memory decay. To overcome these limitations, we adopt a Transformer-Encoder (**Figure 3**) as the expert branch for environment modeling. The input sequence is first linearly projected and enriched with positional encodings to preserve temporal order. Multi-Head layers and Feed Forward Networks are then alternately stacked to capture global correlations across time steps. This design enables efficient parallel computation while extracting long-range temporal patterns. The final output is a compact, context-aware representation of the historical environment, ready for cross-modal fusion.

#### 2.2.2 MobileNetV2 for image representation

RGB images provide a direct window into plant morphology, capturing leaf architecture, canopy compactness, and subtle phenotypic cues associated with overgrowth. However, image data are high-dimensional and computationally demanding, especially in real-world scenarios where efficiency is critical. To balance accuracy with efficiency, we adopt MobileNetV2 as the vision encoder. The MobileNetV2 encoder was initialized with ImageNet-pretrained weights to leverage generic visual features learned from large-scale natural image data. This transfer learning strategy accelerates convergence and improves feature generalization, particularly under the limited size of our strawberry seedling dataset. During fine-tuning, all layers of MobileNetV2 were unfrozen, but we adopted a two-stage training protocol to ensure stable optimization: (1) the first 20 epochs trained only the classifier and fusion layers, keeping the convolutional backbone frozen to adapt the high-level representations to the target domain; (2) the subsequent 80 epochs jointly optimized the entire network with a reduced learning rate (1×10^-4^ for the backbone, 1×10^-^³ for the classifier and fusion modules). The allocation of 20 epochs for the first phase and 80 epochs for the second was determined on convergence stability and prior experience. This two-stage protocol combines two classical transfer learning paradigms—linear probing and fine-tuning (He et al., 2021). The first stage functions as linear probing, during which the pretrained MobileNetV2 weights remain frozen to preserve general visual representations while adapting the classifier and fusion layers to the strawberry seedling domain. The subsequent stage performs full fine-tuning, allowing the entire network to be jointly optimized for better coordination between MobileNetV2, the Transformer encoder, and the fusion module. Prior to encoding, all images are preprocessed through resizing (224×224 px), color normalization, and augmentation to improve generalization. MobileNetV2 employs inverted residual blocks and linear bottlenecks, which reduce computation while maintaining strong representational capacity. This enables the extraction of compact yet discriminative plant features, particularly sensitive to early morphological changes indicative of overgrowth. The resulting feature vector is then forwarded to the cross-modal fusion stage. The detailed structure is outlined in **Table 1**.

**Table 1:** Key architectural specifications of MobileNetV2 used in this study.

Stage	Block type	Output channels (c)	Repeats (n)	Stride(s)
Conv1	3x3 Conv2D	32	1	2
Stage 1	Bottleneck (t=1)	16	1	1
Stage 2	Bottleneck (t=6)	24	2	2
Stage 3	Bottleneck (t=6)	32	3	2
Stage 4	Bottleneck (t=6)	64	4	2
Stage 5	Bottleneck (t=6)	96	3	1
Stage 6	Bottleneck (t=6)	160	3	2
Stage 7	Bottleneck (t=6)	320	1	1
Conv Head	1x1 Conv2D	1280	1	1
Classifier	GAP + FC	k (classes)	1	–

#### 2.2.3 Fusion strategy

In this task, a single-frame image carries rich semantic cues, while the environmental sequence encodes cumulative drivers and precursor signals of overgrowth. Capitalizing on this distinction, we propose a two-stage image-guided Cross-Attention mechanism. Its core principle is to treat the image modality as the dominant query, which selectively attends to the temporal environmental sequence. In doing so, valuable causal signals are highlighted while irrelevant noise is suppressed. The refined environmental context is then fused with the image representation to form a unified decision feature. Intuitively, this image-guided Cross-Attention can be compared to a physician diagnosing a patient: the doctor observes the current symptoms (the plant image) and then searches the patient’s medical history (the environmental time series) to find the most relevant past events that explain the current condition. Similarly, our model uses the visual features of the current image as a “query” to focus on those temporal segments and environmental variables (temperature, PAR, and VWC) most responsible for the observed phenotype. Compared with conventional fusion strategies, this two-stage design enables image-conditioned channel filtering and time-aware retrieval of environmental signals. In particular, when visual evidence is insufficient (e.g., blurred images or early-stage overgrowth), the mechanism adaptively strengthens environmental cues, thereby enhancing the reliability of early recognition.

Formally, the current image feature is defined as the query vector Qimage, which is used to interrogate the historical environment sequence represented by key–value pairs (*K_past_*, *V_past_*). This attention mechanism computes the attention distribution between the image query vector and the time-series key-value pairs using the classic Scaled Dot-Product Attention, generating a context vector, *A_context_*, that incorporates the environmental background, as defined in Equation 1:

(1) $$A_{context}=AttentionQ_{image},K_{past},V_{past}=softmax(\frac{Q_{image}K_{past}^{T}}{\sqrt{d_{k}}})V_{past}$$

Where *d_k_* denotes the key dimension for scaling to stabilize gradients. Through this selective focusing mechanism, only the most relevant historical environment factors are emphasized, enabling the fused representation to carry enhanced causal interpretability.

Finally, to integrate the plant’s instantaneous state (visual modality) with its historical causes (environmental modality), the attention-derived context vector *A_context_* is concatenated with the original image feature vector *F_image_*, followed by a linear projection, as defined in Equation 2:

(2) $$F_{fusion}=Linear(|F_{image}||A_{context}|)$$

**Figure 4:**
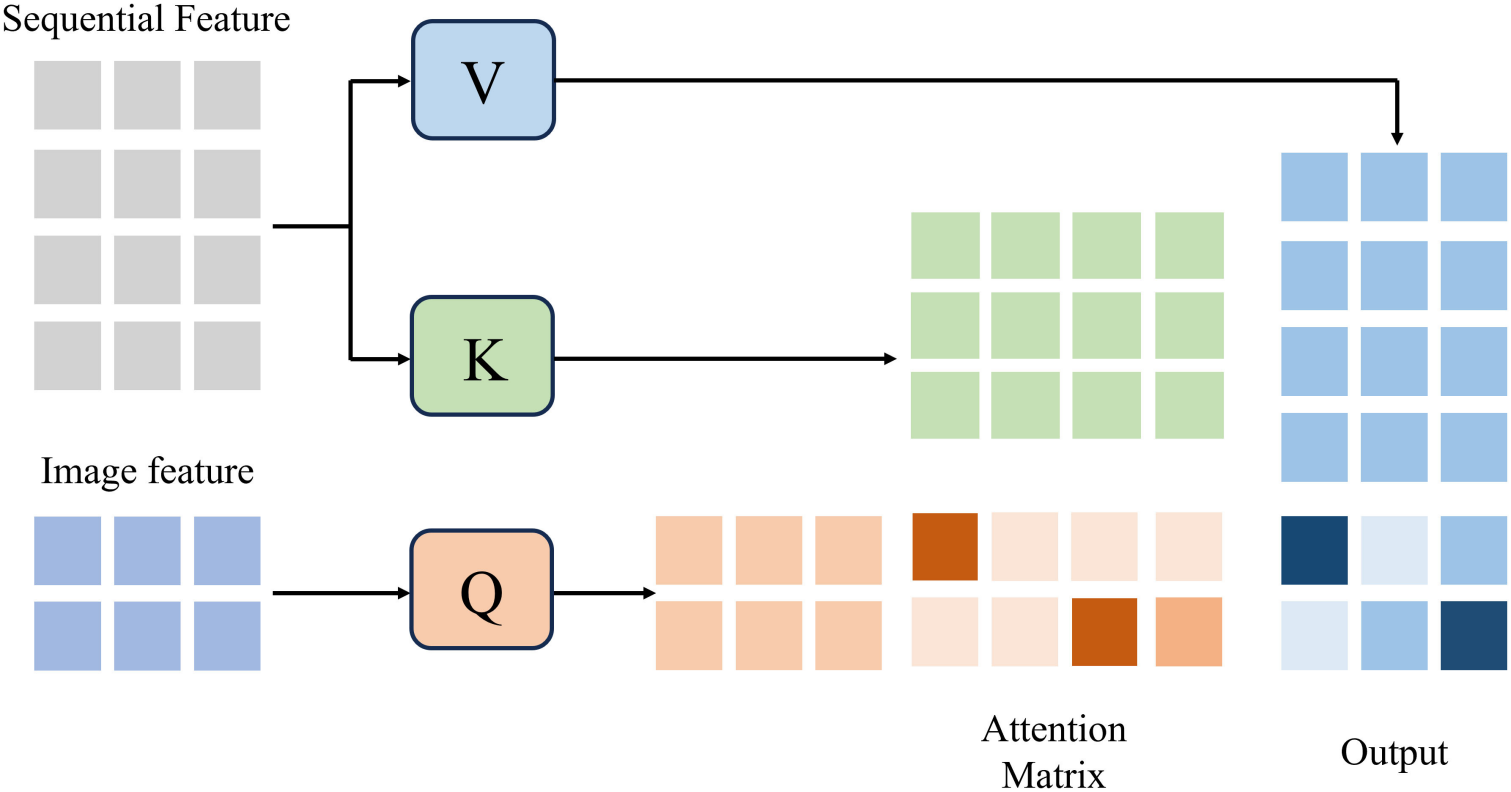
The Cross-Attention module.

The resulting fusion vector *F_fusion_* is then passed into the classifier to output the probability of overgrowth occurrence. **Figure 4** illustrates the overall architecture of the Cross-Attention module.

In the present implementation, the image-guided Cross-Attention employs a multi-head attention mechanism comprising four attention heads and a total embedding dimension of 512, resulting in key and value dimensions of 128 per head. For each head, the image-derived feature vector serves as the Query, while the encoded environmental sequence provides the Key–Value pairs. The outputs from all heads are concatenated and linearly projected to obtain the final fusion representation *F_fusion_*, which is then passed into the classifier.

## 3 Experiments and results

This section systematically evaluates the proposed multimodal Cross-Attention perception network (MM-CAPNet) for detecting overgrowth in strawberry seedlings. We first describe the experimental setup and evaluation metrics, followed by ablation studies to assess the contribution of key components. Visualization analyses are then conducted to interpret model decisions, and finally, MM-CAPNet is compared against multiple baseline and state-of-the-art fusion strategies.

### 3.1 Experimental setup

All experiments were conducted on a high-performance workstation equipped with an Intel Core i9 12900K CPU, 128 GB RAM, and an NVIDIA GeForce RTX 4090 GPU (24 GB). The software environment was Python 3.9 with PyTorch 2.0. Training employed the Adam optimizer (β_1_ = 0.9, β_2_ = 0.999), an initial learning rate of 1×10^−3^, batch size of 32, and a maximum of 100 epochs. Multi-class cross-entropy loss was used, with a dropout rate of 0.3 applied to the multimodal fusion module and classification head. Early stopping was triggered when the validation loss plateaued for 10 consecutive epochs, and the model with the lowest validation loss was retained.

To avoid information leakage, data splits ensured that samples from the same plant (and temporally adjacent windows) did not appear simultaneously in the training, validation, and test sets. All data preprocessing, including augmentation and normalization, was performed after the split, with normalization statistics estimated exclusively from the training set.

For image data, RGB inputs were resized to 224 × 224 pixels, normalized to the range [0, 1], and standardized. Environmental time-series inputs consisted of 120-time steps with three channels, corresponding to temperature, PAR and VWC. Missing values spanning no more than one time step were addressed via linear interpolation, whereas samples containing longer gaps were excluded from the dataset. The dataset was partitioned into training (70%), validation (15%), and test (15%) subsets, ensuring balanced class distributions across each subset.

Model performance was evaluated using the following metrics: Accuracy (ACC): Measures the proportion of correctly classified samples among all test instances, reflecting the overall classification capability of the model. F1-score: The harmonic mean of precision and recall, providing a balanced assessment in the presence of class imbalance. Area Under the ROC Curve (AUC): Quantifies the discriminative ability of the classifier across varying thresholds. The ROC curve illustrates the trade-off between the true positive rate (TPR) and false positive rate (FPR).

These metrics were computed using Equations 3, 4 and 5.

(3) $$ACC=\frac{TP+TN}{TP+FP+TN+FN}\times 100\%$$

(4) $$F1=\frac{2TP}{2TP+FP+FN}\times 100\%$$

(5) $$\begin{array}{c} AUC=\int_{0}^{1}T\text{P}R(FPR)d(FPR),where,\\ TPR=\frac{TP}{TP+FN},FPR=\frac{FP}{FP+TN} \end{array}$$

where true positive (TP), false positive (FP), true negative (TN), and false negative (FN) denote true positives, true negatives, false positives, and false negatives, respectively.

In addition to accuracy-related metrics, the computational efficiency of MM-CAPNet was quantified to support its practical applicability. The complete model contains approximately 5.7 million parameters and requires 1.42 GFLOPs per forward pass. On the workstation equipped with an NVIDIA RTX 4090 GPU, the average inference time per sample was 6.8 ms, while on a standard CPU (Intel i7-11700, 2.5 GHz), the inference time was 94 ms.

### 3.2 Ablation study

The proposed MM-CAPNet integrates two key components: a Cross-Attention based fusion mechanism and modality-specific expert encoders (a Transformer Encoder for temporal sequences and MobileNetV2 for plant imagery). To assess the individual contribution of these components, we conducted an ablation study by systematically removing or replacing them. Four configurations were evaluated: (a) w/o Cross-Attn: removing the Cross-Attention mechanism, fusing features via naive concatenation only; (b) w/o Transformer: excluding the Transformer encoder, discarding temporal data and relying solely on images; (c) w/o MobileNet: excluding the image encoder, discarding visual information and relying solely on temporal sequences; (d) Full Model: complete MM-CAPNet architecture.

As shown in **Table 2**, the complete MM-CAPNet model (d) achieves the highest performance across all evaluation metrics. Removing the Cross-Attention mechanism (a) resulted in accuracy of 82.1%, representing a decrease of 5.5% relative to the full model; the F1-score dropped by 0.054 and AUC by 0.057. This finding suggests that naive concatenation is inadequate for handling the heterogeneity of cross-modal data, as redundant temporal vectors may obscure discriminative image features. In contrast, the Cross-Attention design facilitates dynamic alignment and selective aggregation, allowing visual cues to guide the retrieval of relevant temporal fragments and thereby enhancing multimodal fusion.

**Table 2:** Ablation study results for MM-CAPNet.

Case	Configuration	Accuracy (%)	F1-score	AUC
(a)	without Cross-Attn	82.1	0.818	0.844
(b)	without Transformer	78.5	0.782	0.812
(c)	without MobileNet	72.3	0.719	0.754
(d)	Full MM-CAPNet	87.6	0.872	0.901

Under single-modality conditions, performance declined further. Using only image data (b) yielded accuracy of 78.5%, a reduction of 9.1% compared with the full model. Using only temporal data (c) yielded accuracy of 72.3%, a decrease of 15.3%, representing the lowest performance across all metrics. These results underscore two complementary aspects: (1) the image modality provides dominant discriminative information, directly encoding plant morphology; and (2) the temporal modality remains essential, capturing growth dynamics and environmental cues that cannot be inferred from single snapshots.

Taken together, the ablation study validates the rationality of the MM-CAPNet design: image features provide the primary discriminative basis, temporal sequences contribute historical context, and their synergy, mediated by Cross-Attention, yields the most robust performance.

### 3.3 Visualization and interpretability

**Figure 5:**
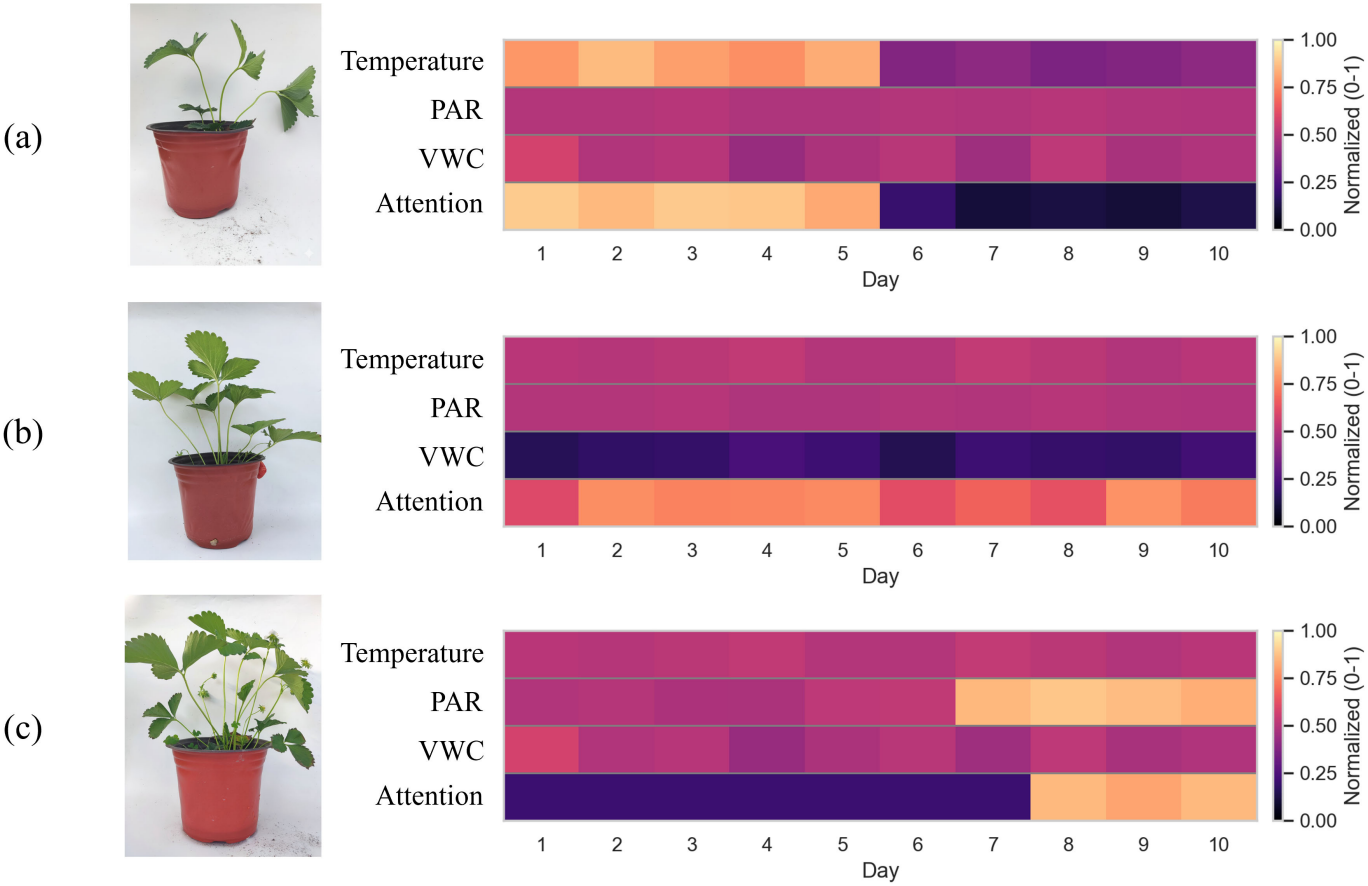
Visualization of cross-modal attention weight distributions under different representative case: **(a)** nighttime high temperature; **(b)** low PAR; **(c)** high VWC.

To interpret the internal decision process of MM-CAPNet, we visualized Cross-Attention weight distributions for three representative samples drawn from the treatment dataset (**Figure 5**), namely: (a) Nighttime High Temperature, (b) Low PAR, and (c) High VWC. These samples were not manually curated but selected as typical cases of the “Overgrowth” category, each reflecting a distinct induced environmental trajectory. Despite their heterogeneity, all cases share the same phenotypic label, enabling us to examine whether the image-guided Cross-Attention module consistently associates visual cues with their corresponding environmental drivers.

In the Nighttime high temperature case (**Figure 5A**), plant images exhibited slightly elongated petioles and dark-green leaves, which are typical signs of overgrowth. When these image features served as the query vector (Qimage), the Cross-Attention module assigned disproportionately high weights to the keys (Kpast) corresponding to the first five days of elevated nighttime temperature, followed by a sharp decline during the recovery phase. This temporal asymmetry suggests that the model does not treat the 10-day sequence as homogeneous input but selectively emphasizes intervals most consistent with the visual phenotype. Importantly, variable-level decomposition revealed that temperature dominated the key–value pairs in these high-weight segments, confirming that the module not only prioritizes when but also which environmental drivers are most relevant.

In the low PAR case (**Figure 5B**), seedling images showed flattened leaves and a loose canopy structure, typical of chronic light suppression. Unlike the transient pattern above, the attention map displayed a more uniform distribution across all 10 days, but with strong intensity concentrated on the PAR channel of the environmental sequence. This indicates that when the stressor is chronic rather than episodic, the Cross-Attention mechanism maintains sustained alignment with the dominant driver while dynamically down-weighting less informative modalities (e.g., humidity and temperature).

In the high VWC case (**Figure 5C**), morphological abnormalities (excessive leaf expansion and canopy loosening) appeared only during the later stages of treatment and were less pronounced. Correspondingly, the Cross-Attention heatmap shifted its focus to the last three days, when humidity levels sharply increased. Interestingly, the weight distribution exhibited a certain delay relative to the humidity surge, suggesting that the model implicitly captured the lagged manifestation of morphological changes in response to environmental perturbations.

In conclusion, the visualization of these representative samples demonstrates that MM-CAPNet has successfully learned deep associations between image phenotypes and environmental–climatic drivers, while exhibiting strong interpretability through its cross-modal attention mechanism. This not only enhances the transparency of the model’s predictions but also indirectly validates the effectiveness of our overgrowth induction experiments in activating typical physiological mechanisms. More importantly, such cross-modal weight responsiveness provides a solid foundation for deploying the model in practical early-warning systems for strawberry seedling vigor.

### 3.4 Performance comparison

To further evaluate the effectiveness of MM-CAPNet in detecting overgrowth, we compared it against several representative multimodal fusion strategies and a published crop-growth prediction model: Early Fusion: direct concatenation of MobileNetV2 (image) and Transformer (temporal) features, followed by classification. Late Fusion: modality-specific encoders followed by self-attention fusion, without explicit guidance of visual dominance. Gated Fusion: adaptive gating to assign weights to each modality, but with limited sensitivity to temporal dynamics. We additionally implemented the CNN-LSTM multimodal fusion framework (Liao et al., 2024). As a classic and high-performing CNN sequence model architecture representative of the agricultural field, we retrained it on our strawberry overgrowth dataset to ensure a fair evaluation. MM-CAPNet: image-centered Cross-Attention mechanism, using visual queries to guide selective retrieval of temporal fragments.

As summarized in **Table 3**, MM-CAPNet consistently achieved the best performance across all evaluation metrics. Its classification accuracy (ACC) reached 87.6%, representing improvements of 6.4%, 4.5%, 3.4%, and 2.7% over Early Fusion, Late Fusion, Gated Fusion, and CNN-LSTM multimodal fusion, respectively. The F1-score reached 0.872, exceeding Early Fusion by 0.066 and CNN-LSTM by 0.031. The AUC reached 0.901, surpassing the next best model by 0.034.

**Table 3:** Overall performance comparison across fusion strategies.

Model	Accuracy (%)	F1-score	AUC
Early Fusion	81.2	0.806	0.829
Late Fusion	83.1	0.824	0.846
Gated Fusion	84.2	0.836	0.861
CNN-LSTM	84.9	0.841	0.867
MM-CAPNet	87.6	0.872	0.901

When evaluating different multimodal fusion strategies, distinct performance differences are observed. Early Fusion, which simply concatenates features from multiple modalities, achieved the lowest classification accuracy, reaching 81.2%. Late Fusion, incorporating attention-based alignment, yielded moderate improvements, with accuracy increasing to 83.1%, although it still lacked deeper semantic interactions between modalities. Gated Fusion introduced adaptive weighting mechanisms across modalities, producing further incremental gains, with accuracy rising to 84.2%. The CNN-LSTM multimodal fusion slightly outperformed Gated Fusion, achieving an accuracy of 84.9%, yet it remained limited in terms of cross-modal interpretability. In contrast, MM-CAPNet, by leveraging an image-guided Cross-Attention mechanism, enabled a more effective semantic alignment between temporal signals and visual cues, thereby achieving the highest overall performance across all three evaluation metrics.

**Figure 6:**
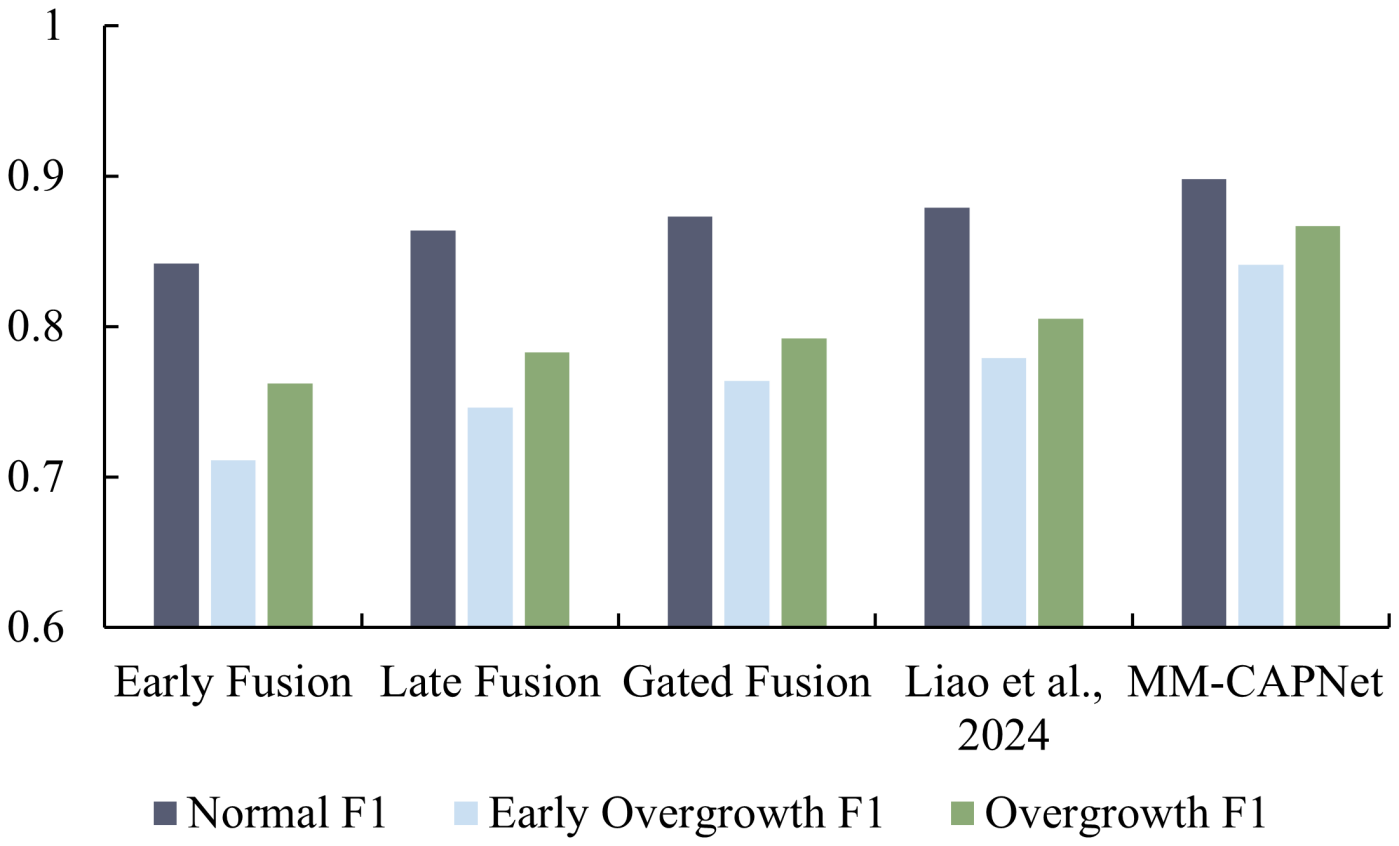
Performance of different models in terms of F1-score across the three categories (Normal, Early Overgrowth, and Overgrowth).

To further reveal model-specific differences in recognizing sparse categories and distinguishing critical phenotypes, we additionally evaluated class-wise F1-score for the three task categories: Normal, Early Overgrowth, and Overgrowth. This analysis, beyond overall metrics, provides a more fine-grained assessment of the model’s discriminative ability across different growth states. The results are illustrated in **Figure 6**.

As shown in the figure, MM-CAPNet consistently achieved the best performance across all categories, with F1-score of 0.898 for Normal, 0.841 for Early Overgrowth, and 0.867 for Overgrowth. The major improvements were concentrated in the two categories related to Overgrowth: compared with Early Fusion, MM-CAPNet yielded gains of 0.130 and 0.105 in the Early Overgrowth and Overgrowth categories, respectively; compared with the next best approach of CNN-LSTM multimodal fusion, the improvements in both categories reached 0.062 and 0.064, reflecting a clear advantage in handling sparse and challenging phenotypes. Even in the majority class (Normal), MM-CAPNet still achieved a net increase of 0.020 over CNN-LSTM, demonstrating that overall improvement was not at the expense of majority-class performance.

Using the average F1-score of the two overgrowth related categories as the critical phenotype index, MM-CAPNet reached 0.854, outperforming CNN-LSTM multimodal fusion, Gated Fusion, Late Fusion, and Early Fusion by 0.062, 0.076, 0.089, and 0.118, respectively. In addition, category level balance was significantly improved: the discrepancy between the Normal category and the average of the two overgrowth categories was reduced from 0.105 in Early Fusion and 0.087 in CNN-LSTM multimodal fusion to only 0.044 in MM-CAPNet, corresponding to a relative reduction of approximately 58% and 49%, respectively.

These findings demonstrate that the image-centered cross-modal attention mechanism enables discriminative visual regions to serve as queries for selectively aggregating semantically related temporal environmental fragments. Consequently, the model not only enhances recognition of both early morphological changes and persistent overgrowth but also improves category balance and overall stability.

## 4 Conclusion

Early warning of overgrowth in strawberry seedlings is essential for fruit quality. At the seedling stage, excessive leaf expansion and stem elongation can delay flower bud initiation, reduce fruit set, and ultimately compromise fruit quality. To address this challenge, the present study constructed a multimodal dataset encompassing three key growth states: Normal, Early Overgrowth, and Overgrowth. Using this dataset, we proposed and validated a cross-temporal multimodal fusion framework, MM-CAPNet. The framework separately models environmental dynamics and visual phenotypes, employing a Transformer encoder for historical environmental sequences and a MobileNetV2 encoder for contemporaneous images. The framework is characterized by an image-guided Cross-Attention mechanism that leverages the current visual phenotype as a query to dynamically retrieve and aggregate the most diagnostically relevant segments from the historical environmental data. This design enables MM-CAPNet to move beyond conventional early fusion or late fusion schemes, achieving a more interpretable and biologically meaningful linkage between observed morphology and its underlying environmental drivers.

Experimental results demonstrated that MM-CAPNet achieved an overall classification accuracy of 87.6%, with corresponding F1-score and AUC values of 0.872 and 0.901, respectively. These results consistently outperformed multiple baseline models and existing fusion strategies. Notably, in the Early Overgrowth class, considered the most confounding phenotype, the proposed method improved the F1-score by an average of approximately 5.3% over the baselines. Furthermore, ablation experiments confirmed the critical role of the Cross-Attention mechanism in enhancing early-stage overgrowth recognition, underscoring the rationality and necessity of the proposed architectural design.

In conclusion, MM-CAPNet provides an effective tool for precise monitoring and regulation of strawberry seedling growth. Although the model achieved strong performance under controlled conditions, its deployment in heterogeneous greenhouse or field environments will require domain adaptation strategies and enhanced image augmentation to accommodate more complex backgrounds. 331humidity may reduce model sensitivity in environments where gas-exchange dynamics or vapor-pressure processes exert stronger physiological influence (e.g., CO_2_-enriched or poorly ventilated greenhouses). This limitation is explicitly acknowledged and will be addressed in future work through the evaluation of their marginal contributions to model performance. Furthermore, the principal contribution of this work lies in enhancing multimodal alignment and early-symptom sensitivity, rather than primarily addressing visual background complexity. Future work will focus on extending the proposed framework to unstructured backgrounds and more heterogeneous environments. Future work will also incorporate periodic calibration and inter-sensor consistency verification to ensure measurement reliability when extending the framework to field-scale or multi-site environments.

## Data Availability

The raw data supporting the conclusions of this article will be made available by the authors, without undue reservation.
